# Higher heart rate variability as a predictor of atrial fibrillation in patients with hypertension

**DOI:** 10.1038/s41598-022-07783-3

**Published:** 2022-03-08

**Authors:** San Ha Kim, Kyoung Ree Lim, Jeong-Hun Seo, Dong Ryeol Ryu, Bong-Ki Lee, Byung-Ryul Cho, Kwang Jin Chun

**Affiliations:** 1 Division of Cardiology, Department of Internal Medicine, Kangwon National University Hospital, Kangwon National University School of Medicine, 156, Baekryung-ro, Chuncheon, Gangwon-do 24289 South Korea; 2 Department of Internal Medicine, Kyung Hee University Hospital at Gangdong, Seoul, South Korea

**Keywords:** Hypertension, Atrial fibrillation

## Abstract

The autonomic nervous system (ANS) plays an important role in the initiation and maintenance of atrial fibrillation (AF). However, the meaning of higher heart rate variability (HRV) in predicting AF remains unclear. Among 2100 patients in the Holter registry, a total of 782 hypertensive patients were included in this study. Baseline HRV was measured by time domain and frequency domain methods using 24-h Holter monitoring. The primary outcome was the development of AF. During an average follow-up of 1.1 years, 44 patients developed AF. Higher HRV parameters including high-frequency (*P* < 0.001), the square root of the mean squared differences of successive NN intervals (*P* < 0.001), and the percentage of NN intervals that are more than 50 ms different from the previous interval (*P* < 0.001) were associated with the occurrence of AF in univariate analysis. Premature atrial contractions burden, lower baseline heart rate, age, hemodialysis, coronary artery disease, and chronic heart failure were also associated with AF. In Cox regression analysis, higher HRV (representing excessive autonomic fluctuation) was an independent risk factor for AF. Excessive autonomic fluctuation represented by higher HRV in patients with hypertension was associated with an increased risk of AF.

## Introduction

Atrial fibrillation (AF) is the most common cardiac arrhythmia requiring medical therapy^1^. Various pathophysiological mechanisms for the development of AF have been studied^2^. Recently, there has been an increasing evidence that the dysfunction of the autonomic nervous system (ANS) including the sympathetic and parasympathetic nervous systems, and the interaction between sympathetic and parasympathetic nervous systems are involved in the pathogenesis of AF^3^.

Despite the increasing evidence of an association between dysfunction of ANS and AF, whether abnormalities in the ANS can predict the development of AF remains unclear. Several population-based studies showed that lower heart rate variability (HRV) was associated with an increased risk of new-onset AF^4–6^. However, another study reported that higher HRV value was associated with the incident AF^7^. The electrocardiography (ECG) recording time used to analyze HRV and analysis methods differed among the studies, leading to inconsistent results.

Hypertension is the most common cardiovascular risk factor in patients with AF^8^. Previous studies reported that HRV was not only associated with cerebrovascular diseases such as stroke and myocardial infarction but also relatively mild disease such as hypertension and anxiety disorders^9–11^. The objective of this study was to investigate whether HRV using 24-h Holter monitoring could predict the development of AF in patients with hypertension.

## Methods

### Study population

The study population was selected from the Kangwon National University Holter registry. A total of 2100 patients underwent 24-h Holter monitoring between May 2018 and April 2019. Patients were eligible for this study if they were in sinus rhythm at baseline 24-h Holter monitoring and had HRV data. The exclusion criteria were: (1) < 18 years of age, (2) had persistent AF or paroxysmal AF lasting more than 30 s at baseline 24-h Holter monitoring, (3) had temporary or permanent pacemaker, (4) had complete atrioventricular block, or (5) < 22 h of recording. After excluding 480 patients who met the exclusion criteria, 1620 patients were identified. Among the 1620 patients, 782 patients with hypertension were finally analyzed.

This retrospective observational cohort study was conducted in accordance with the principles of the Declaration of Helsinki. The Institutional Review Board of Kangwon National University Hospital approved the study protocol (KNUH-2020-04-021-001). The need for informed consent was waived for this retrospective study by the Institutional Review Board of Kangwon National University Hospital. This study followed the Strengthening the Reporting of Observational Studies in Epidemiology (STROBE) reporting guidelines.

### Study design and data collection

Baseline demographic data, cardiovascular risk factors, medications, and clinical outcomes were retrospectively analyzed by reviewing the medical records. The study population was divided into two groups according to the presence of hypertension. Holter data including the HRV parameters and cardiovascular risk factors were compared. Whether HRV parameters could predict the occurrence of AF during the follow-up period in patients with hypertension was also determined.

### Study outcome

The study outcome was the development of AF during follow-up. The development of AF was defined as a standard 12-lead ECG recording or Holter recording with ≥ 30 s of AF. The development of AF was evaluated by reviewing the medical records from our hospital. All ECG and 24-h Holter monitoring data were interpreted by one cardiologist.

### Heart rate variability

HRV data were acquired by 24-h Holter monitoring (MARS, GE Healthcare, Chicago, Illinois, United States) and measured by frequency domain and time domain methods. Fast Fourier Transform (FFT) (a non-parametric method of spectral estimation) was used to convert single-lead ECG signals to power spectral densities. The cubic spline-interpolated NN interval function was sampled at 1024 samples/300 s or 3.413 samples/s. NN interval ratios of < 0.80 or > 1.20 and NN intervals of < 150 ms or > 5000 ms were excluded prior to HRV analysis. The power spectral density included very low-frequency (VLF) component (0.0033–0.0400 Hz), low-frequency (LF) component (0.0400–0.1500 Hz), high-frequency (HF) component (0.1500–0.4000 Hz), and the ratio of two components (LF/HF ratio). The time domain methods included the standard deviation of the NN interval (SDNN), the standard deviation of all 5-min average NN interval (SDANN), the average standard deviation of all 5-min NN interval (ASDNN), the square root of the mean squared differences of successive NN intervals (rMSSD), the percentage of NN intervals that are more than 50 ms different from the previous interval (pNN50), and the count of intervals that were more than 50 ms different from the previous interval (BB50).

### Statistics

Continuous variables are expressed as mean ± standard deviation or median and interquartile range. Categorical variables are expressed as frequency and percentage. To evaluate the difference in HRV values and clinical characteristics according to the occurrence of AF, Student’s unpaired t test was used for normally distributed data and Mann–Whitney test was used for skewed data. Categorical variables were analyzed with chi-square test or Fisher’s exact test. Receiver operating characteristic (ROC) curve analysis was used to select the cut-off value between the HRV parameter and the occurrence of AF. Cox regression analysis was used to calculate hazard ratios (HRs) and 95% confidence intervals (CIs) for the risk factors associated with the occurrence of AF. Calculations were performed using the Statistical Package for the Social Sciences version 20.0 for Windows (IBM Corp., Armonk, NY, USA). A *P*-value less than 0.05 was considered statistically significant.

## Results

**Figure 1:**
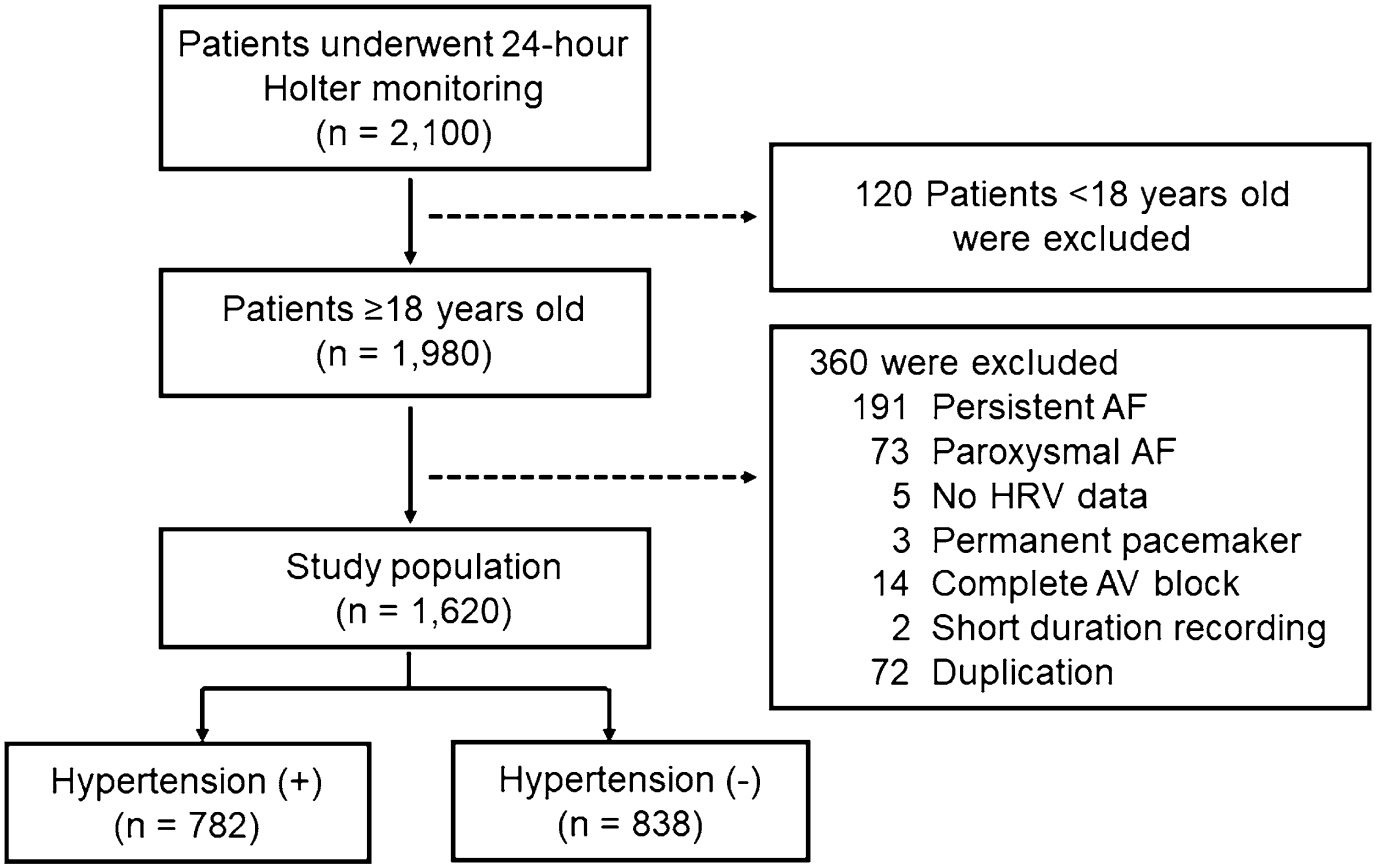
Enrollment of patients in the study. AF, atrial fibrillation; HRV, heart rate variability.

Among the 2100 patients who underwent 24-h Holter monitoring between May 2018 and April 2019, 480 patients were excluded due to the following: 120 patients were younger than 18 years old, 191 patients had persistent AF, 73 patients had paroxysmal AF, 5 patients had no HRV data, 3 patients had permanent pacemaker, 14 patients had complete atrioventricular block, 2 patients had insufficient recording, and 72 patients performed Holter monitoring repeatedly (Fig. 1). After excluding 480 patients who met the exclusion criteria and 838 patients without hypertension, 782 patients with hypertension were finally analyzed.

### Baseline clinical characteristics

The baseline clinical characteristics of the study population are shown in Table 1. The baseline characteristics and cardiovascular risk factors according to the occurrence of AF in patients with hypertension are shown in Table 2. The patients with AF occurrence were older, with more end stage kidney disease on dialysis, coronary artery disease, chronic heart failure, and history of AF. However, there was no significant difference in gender, diabetes, or history of stroke between the two groups.

**Table 1:** Baseline characteristics of study population.

	Hypertensive patients (n = 782)
Age (years)	69.8 ± 12.3
Male (n, %)	415 (53.1)
DM (n, %)	240 (30.7)
CKD (n, %)	50 (6.4)
Hemodialysis (n, %)	12 (1.5)
Dyslipidemia (n, %)	435 (55.7)
CAD (n, %)	107 (13.7)
History of CVA (n, %)	113 (14.5)
Acute CVA (n, %)	253 (32.4)
Chronic HF (n, %)	34 (4.3)
Acute HF (n, %)	11 (1.4)
History of AF (n, %)	66 (8.4)

DM, diabetes mellitus; CKD, chronic kidney disease; CAD, coronary artery disease; CVA, cerebrovascular accident; HF, heart failure; AF, atrial fibrillation.

**Table 2:** Clinical characteristics according to the occurrence of atrial fibrillation in patients with hypertension.

	AF occurrence (+) (n = 44)	No AF occurrence (n = 738)	*P* value
Age (years)	75.5 ± 10.2	69.5 ± 12.4	0.001
Male (n, %)	25 (56.8)	390 (52.8)	0.608
DM (n, %)	17 (38.6)	223 (30.2)	0.239
CKD (n, %)	4 (9.1)	46 (6.2)	0.452
Hemodialysis (n, %)	3 (6.8)	9 (1.2)	0.003
Dyslipidemia (n, %)	25 (56.8)	410 (55.6)	0.927
CAD (n, %)	12 (27.3)	95 (12.9)	0.007
History of CVA (n, %)	9 (20.5)	104 (14.1)	0.244
Acute CVA (n, %)	10 (22.7)	243 (32.9)	0.160
Chronic HF (n, %)	5 (11.4)	29 (3.9)	0.019
Acute HF (n, %)	2 (4.5)	9 (1.2)	0.069
History of AF (n, %)	20 (45.5)	46 (6.2)	< 0.001

AF, atrial fibrillation; DM, diabetes mellitus; CKD, chronic kidney disease; CAD, coronary artery disease; CVA, cerebrovascular accident; HF, heart failure.

### Heart rate variability and Holter data

LF and HF component in the frequency domain method and ASDNN, rMSSD, pNN50, and BB50 in the time domain method were higher in the AF occurrence group (Table 3). Mean heart rate was faster in the no AF occurrence group (*P* = 0.002). However, the burden of premature atrial contractions (PAC) was significantly higher in AF occurrence group (*P* < 0.001).

**Table 3:** Differences in heart rate variability and Holter data according to the occurrence of atrial fibrillation in patients with hypertension.

	AF occurrence (+) (n = 44)	No AF occurrence (n = 738)	*P* value
VLF (ms)	30.3 ± 20.4	23.8 ± 12.0	0.075
LF (ms)	20.1 ± 16.3	13.9 ± 9.0	0.011
HF (ms)	15.3 ± 7.7	10.7 ± 5.9	< 0.001
LF/HF ratio	1.23 ± 0.40	1.45 ± 3.18	0.098
SDNN (ms)	117.9 ± 72.2	108.0 ± 40.1	0.848
SDANN (ms)	98.9 ± 59.3	96.1 ± 38.6	0.536
ASDNN (ms)	56.4 ± 33.1	43.5 ± 18.8	0.021
rMSSD (ms)	40.4 ± 21.0	28.0 ± 13.1	< 0.001
pNN50 (%)	15.5 ± 14.2	8.0 ± 8.3	< 0.001
BB50 (beats)	10,070 ± 7423	6810 ± 6400	< 0.001
Mean heart rate (bpm for 24 h)	66 (54–77)	71 (64–80)	0.002
Premature atrial contractions (beats for 24 h)	512 (55–4561)	56 (21–311)	< 0.001

VFL, very low frequency; LF, low frequency; HF, high frequency; SDNN, standard deviation of the NN interval; SDANN, standard deviation of all 5-min mean NN interval; ASDNN, average standard deviation of all 5-min RR intervals; rMSSD, square root of the mean squared differences of successive NN interval; pNN50, the percentage of RR intervals that are more than 50 ms different from the previous interval; BB50, the count of intervals that are more than 50 ms different from the previous interval.

### Risk factors for AF occurrence

During an average follow-up of 1.1 years, 44 patients developed AF. Among the 44 patients who developed AF during the follow-up period, 27 (61.4%) patients were found to have paroxysmal AF and 17 (38.6%) patients were found to have persistent AF at the initial AF identification. Of the 27 patients found to have paroxysmal AF, 10 patients had a history of AF, and 10 of the 17 patients found to have persistent AF had a history of AF.

**Figure 2:**
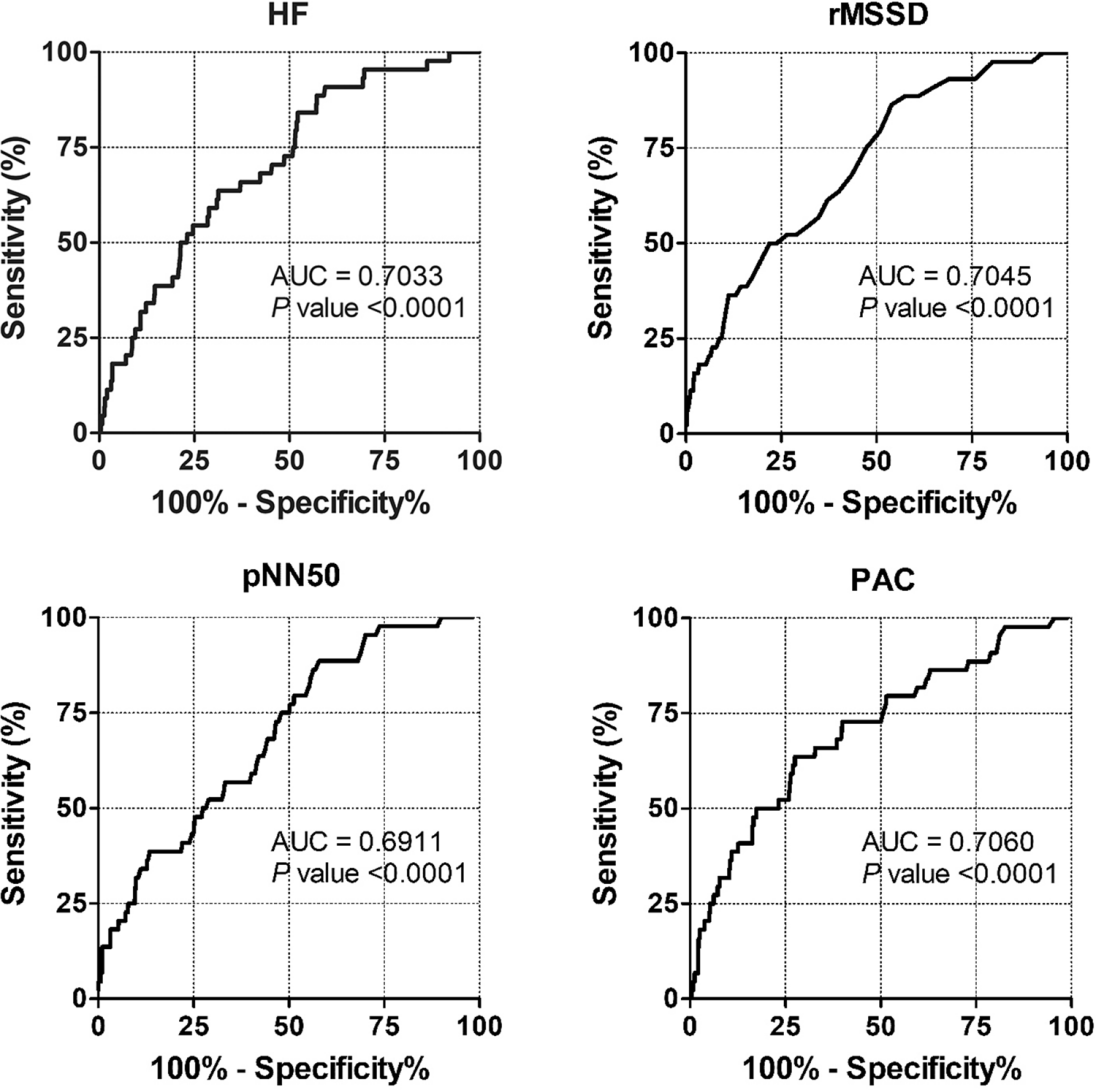
ROC curve of heart rate variability and premature atrial contractions to predict the occurrence of atrial fibrillation. ROC, Receiver operating characteristic; HF, high frequency; rMSSD, square root of the mean squared differences of successive NN interval; pNN50, the percentage of RR intervals more than 50 ms different from the previous interval; PAC, premature atrial contraction; AUC, area under the curve.

**Figure 3:**
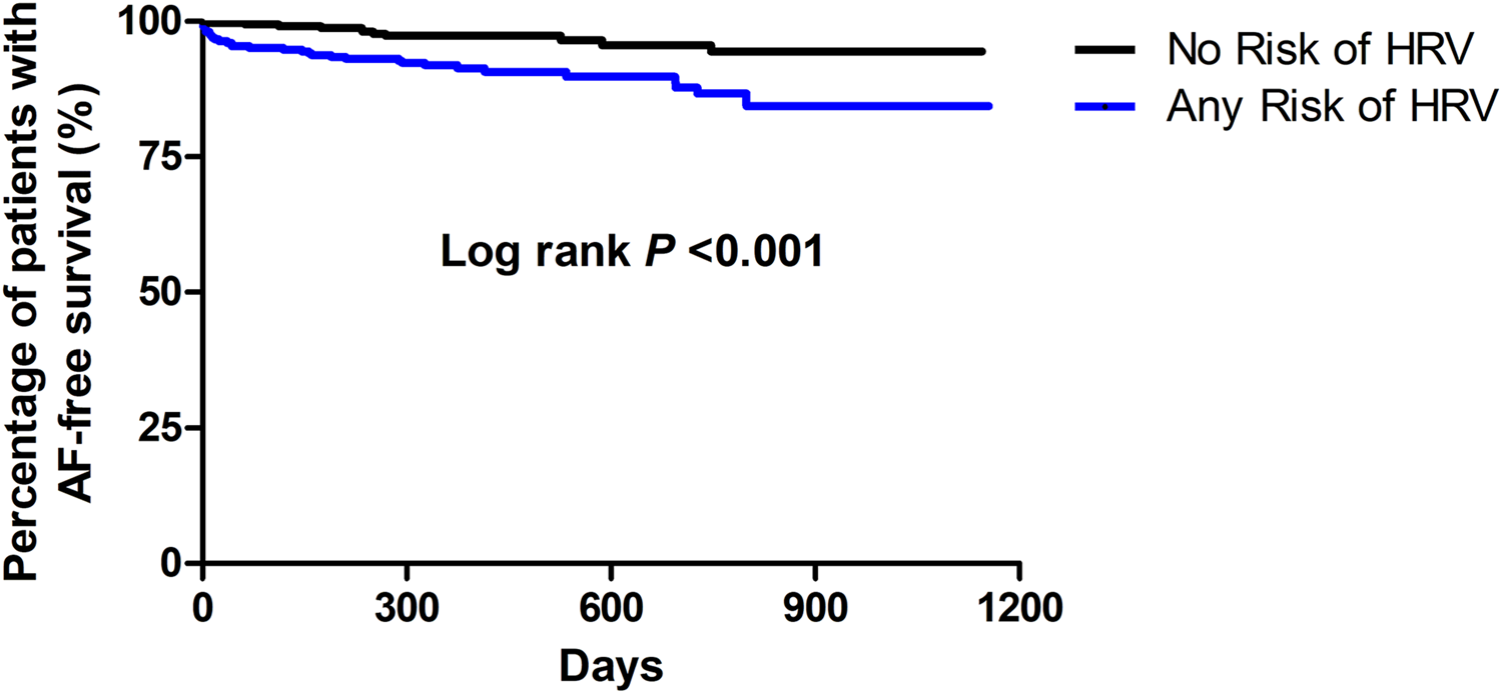
Kaplan–Meier estimate of AF-free survival according to the presence of any risk of HRV. AF, atrial fibrillation; HRV, heart rate variability. Any risk of HRV meant that at least one of the abnormal HRV parameters (HF, rMSSD, and pNN50) were present. AF, atrial fibrillation; HRV, heart rate variability.

The univariate analysis showed that the traditional risk factors such as age, coronary artery disease, hemodialysis, and chronic heart failure were associated with an increased risk of AF (Table 4). Several HRV parameters including VLF (*P* = 0.002), LF (*P* < 0.001), HF (*P* < 0.001), rMSSD (*P* < 0.001), and pNN50 (*P* < 0.001) were associated with AF occurrence. History of AF, the mean heart rate, and PAC count was also associated with AF occurrence. ROC analysis for HF, rMSSD, pNN50, and PAC count as predictors of AF occurrence revealed areas under the curve of 0.7033, 0.7045, 0.6911, and 0.7060, respectively (all *P* < 0.001, Fig. 2). The best HF cut off value of 11.1 for AF occurrence resulted in a sensitivity of 65.9% and a specificity of 62.9%. The sensitivity and specificity of the rMSSD at a cut off value of 29.5 for AF occurrence were 61.4% and 62.9%, respectively. In the case of pNN50 at a cut off value of 7.0, the sensitivity and specificity were 61.4% and 58.5%, respectively. Satisfying any one of these three HRV cut off values was associated with the occurrence of AF (*P* < 0.001, Table 5). The Kaplan–Meier estimates of AF occurrence according to any risk of HRV parameters are presented in Fig. 3 (log rank *P* < 0.001). Cox regression analysis showed that older age, higher PAC burden, hemodialysis, coronary artery disease, history of AF, and any risk of HRV parameters were independent predictors for the occurrence of AF (Table 6).

**Table 4:** Univariate analysis of the occurrence of atrial fibrillation.

Variable	HR (95% CI)	P value
Age	1.050 (1.018–1.082)	0.002
Female gender	0.841 (0.463–1.527)	0.569
DM	1.490 (0.812–2.733)	0.198
CAD	2.219 (1.142–4.309)	0.019
CKD	1.540 (0.550–4.310)	0.411
Hemodialysis	6.298 (1.934–20.057)	0.002
History of CVA	1.467 (0.705–3.054)	0.306
Acute CVA	0.622 (0.307–1.260)	0.188
Chronic heart failure	2.700 (1.064–6.853)	0.037
Acute heart failure	3.257 (0.788–13.464)	0.103
History of AF	9.921 (5.478–17.969)	< 0.001
VLF	1.019 (1.007–1.032)	0.002
LF	1.031 (1.015–1.047)	< 0.001
HF	1.076 (1.044–1.108)	< 0.001
LF/HF ratio	0.509 (0.242–1.070)	0.075
SDNN	1.004 (0.998–1.010)	0.208
ASDNN	1.017 (1.008–1.025)	< 0.001
rMSSD	1.036 (1.023–1.049)	< 0.001
pNN50	1.057 (1.035–1.080)	< 0.001
BB50	1.000 (1.000–1.000)	0.003
Mean heart rate	0.960 (0.935–0.986)	0.003
PAC counts	1.000 (1.000–1.000)	< 0.001

DM, diabetes mellitus; CKD, chronic kidney disease; CAD, coronary artery disease; CVA, cerebrovascular accident; VLF, very low frequency; LF, low frequency; HF, high frequency; SDNN, standard deviation of the NN interval; ASDNN, average standard deviation of all 5-min RR intervals; rMSSD, square root of the mean squared differences of successive NN interval; pNN50, the percentage of RR intervals that are more than 50 ms different from the previous interval; BB50, the count of intervals that are more than 50 ms different from the previous interval.

**Table 5:** Relationship between HRV risk score according to cut–off value and occurrence of atrial fibrillation.

Variable	HR (95% CI)	*P* value
HF ≥ 11.1 (ms)	3.084 (1.653–5.753)	< 0.001
rMSSD ≥ 29.5 (ms)	2.528 (1.378–4.638)	0.003
pNN50 ≥ 7.0 (%)	2.261 (1.223–4.179)	0.009
One or more risk of HRV^a^	3.261 (1.648–6.452)	0.001
**Risk score of HRV**^**b**^		
0 (n = 405)	1.0 (reference)	
1 (n = 65)	4.249 (1.647–10.963)	0.003
2 (n = 54)	0.688 (0.086–5.173)	0.699
3 (n = 258)	3.597 (1.770–7.310)	< 0.001
PACs ≥ 145 beats for 24 h	3.788 (2.031–7.068)	< 0.001

HRV, heart rate variability; HF, high frequency; rMSSD, square root of the mean squared differences of successive NN interval; pNN50, the percentage of RR intervals that are more than 50 ms different from the previous interval; PAC, premature atrial contraction.

^a^Indicates that at least one of the above mentioned abnormal HRV parameters (HF, rMSSD, and pNN50) were present.

^b^Sum of the above mentioned abnormal HRV parameter (HF, rMSSD, and pNN50).

**Table 6:** Multivariate analysis of the occurrence of atrial fibrillation.

Variable	HR (95% CI)	P value
Age	1.032 (1.001–1.065)	0.043
Female gender	0.667 (0.359–1.240)	0.201
PAC counts	1.000 (1.000–1.000)	< 0.001
Hemodialysis	3.853 (1.130–13.139)	0.031
CAD	2.074 (1.015–4.241)	0.045
Chronic heart failure	0.710 (0.261–1.932)	0.502
History of AF	7.738 (4.202–14.250)	< 0.001
One or more risk of HRV^a^	2.396 (1.167–4.919)	0.017

PAC, premature atrial contraction; CAD, coronary artery disease; HRV, heart rate variability.

^a^Indicates that at least one of the abnormal HRV parameters (HF, rMSSD, and pNN50) were present.

## Discussion

The main finding of this study was that higher HRV in patients with hypertension could predict the development of AF independent of demographics or known cardiovascular risk factors. HF, rMSSD, and pNN50 were notably associated with the occurrence of AF, along with age, hemodialysis, and high PAC burden, which are traditional risk factors for AF. Our findings suggest that higher HRV representing abnormal autonomic fluctuation is associated with a higher risk of AF development. Separately, patients with hypertension had more comorbidities and lower HRV than those without hypertension.

The ANS plays an important role in the initiation and maintenance of AF through atrial electrical remodeling^12,13^. A previous study showed that increased vagal activity could cause shortening of the atrial effective refractory periods^12^. Another study revealed that AF could be initiated by premature beats during vagal stimulation^14^. Moreover, autonomic fluctuations preceding AF are common^15,16^. Most studies evaluating these phenomena used HRV parameters to estimate dysfunction of ANS^15,16^. HRV is a quantitative method used to measure the balance between the sympathetic and parasympathetic nervous system^17,18^. HRV indicates fluctuations in autonomic inputs to the heart rather than the mean level of autonomic tones. Therefore, not only lower HRV values, but also higher HRV values might indicate abnormal conditions.

Two previous long-term follow-up studies showed that lower HRV was associated with an increased risk of AF^4,5^. However, another study reported that the association between lower SDNN and incident AF was not significant after adjusting for traditional risks factor for AF^6^. One recently published study showed that not only lower HRV but also higher rMSSD was associated with the development of AF^7^. These inconsistent results might be due to differences in ECG recording time and the study population used in HRV analysis. In our study, higher HRV parameters were associated with the development of AF, contrary to the results of some previous studies. There are several possible explanations for these differences. First, our study focused on hypertensive patients, unlike previous studies using general population^4–6^. Hypertensive patients generally have lower HRV than healthy people^10^. In our study, patients with hypertension had more comorbidities and lower HRV, consistent with other studies (Supplementary Tables S1 and S2). Since the mean HRV value might be higher in the general population than in hypertensive patients, higher HRV values might not be significantly different enough to predict AF. Second, we used 24-h Holter monitoring to measure HRV. In general, the time domain methods are ideal for long-term ECG recording, whereas the frequency domain methods are preferred to the time domain methods for short-term recording^17^. In the two previous long-term follow-up studies, short-term (2-min^4^ and 45-min^5^) ECG recording was used. Therefore, the HRV parameters measured by time domain methods were more appropriate in our study. Third, a closer look at the previous Atherosclerosis Risk in Communities (ARIC) cohort study reported that lower HRV was associated with an increased risk of AF, a figure in that report showed that higher HRV values were also associated with the occurrence of AF. However, this association between higher HRV values and the occurrence of AF was not addressed in previous ARIC cohort study^4^.

Parasympathetic dominance is associated with an increased propensity for AF, and vagal-mediated paroxysmal AF is preferentially seen in young individuals who have structurally normal heart^19^. Generally, HF components are predominantly modulated by the parasympathetic nervous system, whereas LF components are considered a marker of sympathetic modulation^20,21^. HRV represents the degree of autonomic fluctuations rather than the mean level of autonomic tones. Therefore, relatively higher HRV could be considered excessive fluctuations in the ANS rather than a physiologic condition. In other words, even though hypertensive patients generally had low HRV than those without hypertension, hypertensive patients with comparatively higher HRV might be considered to have disproportionate fluctuations in the ANS. A previous study^7^ showed that higher rMSSD was associated with incident AF could be interpreted from this point of view.

We enrolled patients with sinus rhythm at baseline 24-h Holter monitoring. Therefore, patients with paroxysmal AF were enrolled in this study. Sixty-six of 782 (8.4%) hypertensive patients had a history of AF. Perhaps more patients with a history of AF might be included due to its asymptomatic feature. Among 66 patients with a history of AF, AF recurred in 20 patients. A history of AF was an independent risk factor for AF occurrence as well as higher HRV in this study. When we examine a patient in an outpatient clinic, it is difficult to know whether the patient has a history of AF. Therefore, it is important to predict the occurrence of AF regardless of a history of AF, and the HRV value would be one of the factors that could predict the occurrence of AF.

This study had several limitations. First, this was a retrospective cohort study. We could not fully evaluate many other risk factors for AF, including obstructive sleep apnea, body weight, alcohol consumption, or physical activity. Second, the duration of follow-up was relatively short and the degree of effort to identify AF varied from patient to patient. Since most patients with AF are asymptomatic, it is difficult to detect AF with an intermittent ECG test. Regular 24-h Holter monitoring could not be performed due to the limitations of the retrospective study. Therefore, AF incidence in our study was low. Third, our study enrolled patients undergoing hemodialysis. Because blood pressure and autonomic tone change before and after dialysis, short-term ECG recordings are generally preferred to avoid environmental influences. However, since the number of dialysis patients in our study was very small, we analyzed HRV data using 24-h Holter recordings. Finally, since HRV parameters measured by frequency domain methods were made by 24-h Holter recording, there might be a problem of stationarity.

In conclusion, higher HF, rMSSD, and pNN50 in patients with hypertension as surrogate markers for excessive autonomic fluctuation could predict the occurrence of AF. Frequent AF screening should be considered for hypertensive patients with higher HRV values and other risk factors for AF. Further large scale, prospective studies are needed to verify our findings.

## Supplementary Information

Supplementary Tables.

## Data Availability

The datasets used and/or analyzed in the present study can be shared on reasonable request.
