# Supplementary material for: Higher heart rate variability as a predictor of atrial fibrillation in patients with hypertension
Source: Sci Rep. 2022 Mar 8;12:3702. doi: 10.1038/s41598-022-07783-3 (PMC8904557; doi:10.1038/s41598-022-07783-3)
Supplement: Supplementary file 1 — Supplementary Tables. [file 41598_2022_7783_MOESM1_ESM.pdf]

Higher Heart Rate Variability as a Predictor of Atrial fibrillation in Patients with Hypertension

San Ha Kim[1], Kyoung Ree Lim[2], Jeong-Hun Seo[1], Dong Ryeol Ryu[1], Bong-Ki Lee[1], Byung-Ryul Cho[1], Kwang Jin Chun[1]*

[1]Division of Cardiology, Department of Internal Medicine, Kangwon National University Hospital, Kangwon National University School of Medicine, Chuncheon, South Korea

[2]Department of Internal Medicine, Kyung Hee University Hospital at Gangdong, Seoul, South Korea

San Ha Kim and Kyoung Ree Lim contributed equally to this work

Address for correspondence*

Kwang Jin Chun, MD, PhD, Division of Cardiology, Department of Internal Medicine, Kangwon National University Hospital, 156, Baekryung-ro, Chuncheon, Gangwon-do, 24289, South Korea. Tel: +82-33-258-9155; Fax: +82-33-258-2432; E-mail: imchunn@naver.com

Supplementary Information

Supplementary Table S1. Baseline characteristics of study populations according to the presence of hypertension

	Hypertension (+)<br>(n=782)	Hypertension (-)<br>(n=838)	*P* value
Age (years)	69.8 ± 12.3	55.6 ± 18.8	<0.001
Male (n, %)	415 (53.1)	408 (48.7)	0.078
DM (n, %)	240 (30.7)	87 (10.4)	<0.001
CKD (n, %)	50 (6.4)	4 (0.5)	<0.001
Hemodialysis (n, %)	12 (1.5)	1 (0.1)	0.001
Dyslipidemia (n, %)	435 (55.7)	122 (14.6)	<0.001
CAD (n, %)	107 (13.7)	28 (3.3)	<0.001
History of CVA (n, %)	113 (14.5)	44 (5.3)	<0.001
Acute CVA (n, %)	253 (32.4)	133 (15.9)	<0.001
Chronic HF (n, %)	34 (4.3)	10 (1.2)	<0.001
Acute HF (n, %)	11 (1.4)	5 (0.6)	0.099
History of AF (n, %)	66 (8.4)	35 (4.2)	<0.001

DM, diabetes mellitus; CKD, chronic kidney disease; CAD, coronary artery disease; CVA, cerebrovascular accident; HF, heart failure; AF, atrial fibrillation

Supplementary Table S2. Heart rate variability and Holter data according to the presence of hypertension

	Hypertension (+) (n=782)	Hypertension (-) (n=838)	*P* value
VLF (ms)	24.1 ± 12.7	29.7 ± 31.5	<0.001
LF (ms)	14.3 ± 9.7	17.9 ± 9.8	<0.001
HF (ms)	10.9 ± 6.1	12.4 ± 6.4	<0.001
LF/HF ratio	1.33 ± 0.44	1.49 ± 0.48	<0.001
SDNN (ms)	108.5 ± 42.6	126.9 ± 41.3	<0.001
SDANN (ms)	96.2 ± 40.0	113.4 ± 41.2	<0.001
ASDNN (ms)	44.2 ± 20.1	51.6 ± 19.5	<0.001
rMSSD (ms)	28.7 ± 13.9	31.1 ± 13.3	<0.001
pNN50 (%)	8.5 ± 8.9	10.2 ± 9.3	<0.001
BB50 (beats)	6994 ± 6501	8994 ± 7554	<0.001
Mean heart rate (bpm for 24 hours)	71 (64 – 80)	73 (66 – 79)	0.130
Premature atrial contractions (beats for 24 hours)	59 (22 – 362)	27 (6 – 115)	<0.001

VFL, very low frequency; LF, low frequency; HF, high frequency; SDNN, standard deviation of the NN interval; SDANN, standard deviation of all 5-min mean NN interval; ASDNN, average standard deviation of all 5-min RR intervals; rMSSD, square root of the mean squared differences of successive NN interval; pNN50, the percentage of RR intervals that are more than 50 ms different from the previous interval; BB50, the count of intervals that are more than 50 ms different from the previous interval
